# The Passive Contact Stability of Blue Sheep Hoof Based on Structure, Mechanical Properties, and Surface Morphology

**DOI:** 10.3389/fbioe.2020.00363

**Published:** 2020-04-24

**Authors:** Hailin Kui, Xiangyu Liu, Jing Liu, Wei Liang, Shiwu Zhang, Zhihui Qian, Lei Ren

**Affiliations:** ^1^College of Transportation, Jilin University, Changchun, China; ^2^Key Laboratory of Bionic Engineering, Jilin University, Changchun, China; ^3^Department of Precision Machinery and Precision Instrumentation, University of Science and Technology of China, Hefei, China; ^4^School of Mechanical, Aerospace and Civil Engineering, University of Manchester, Manchester, United Kingdom

**Keywords:** blue sheep, biological composite, keratin, hoof, contact stability

## Abstract

As the only component that contacts the ground and rock, the hooves of blue sheep may play a crucial role in their excellent climbing abilities. In this study, we used a combination of techniques, including scanning electron microscopy, infrared spectroscopy and nanoindentation, to characterize the surface morphology, structure, material composition, and mechanical properties of blue sheep hoof and investigate the potential contributions of these properties to the establishment of passive contact stability. Straight and curled microscopic lamellar morphology were found on the hoof surfaces. The cross section of the hoof revealed four layers, and each layer had a unique structure. Finite element analysis was employed to verify that the surface morphology and microstructure effectively contributed to the slip resistance and impact cushioning, respectively. Analyses of the energy and infrared spectra showed that the organic and inorganic substances in different regions of the hoof had similar components but different contents of those components. The hoof was mainly composed of keratin. From the outside to the inside, gradients in both the modulus and hardness were observed. These factors help the hoof alleviate high impact strengths and increase contact stability. These findings further our understanding of the unique mechanism of blue sheep hoof and may help in the development of novel biomimetic materials and mechanical components with enhanced friction and contact stability properties.

## Introduction

Terrestrial animals have developed complex feet to enable dynamic locomotion in different environments (Woodward and Sitti, 2018). Blue sheep (*Pseudois nayaur*), a relatively widely distributed mountain ungulate, have cloven hooves and an excellent ability to scamper up precipitous cliffs (even when the slope is ∼90°) (Luo, 2011; Zhang, 2011). As the only body component in contact with the ground, hooves should play multiple crucial roles, such as attenuating impact with the ground, supporting gravity, maintaining locomotor stability, and transmitting or generating propulsive power during locomotion (Ker et al., 1987; Carrier et al., 1994).

Over the past few decades, a large number of studies have been conducted on a variety of topics related to blue sheep, such as determining the effects of human activity on blue sheep (Jiang et al., 2013), investigating cardiopulmonary hemodynamics (Sakai et al., 2003), detecting viruses (Bao et al., 2011), assessing genetic diversity (Yang et al., 2015), and detecting DNA (Liu et al., 2015) in blue sheep as well as determining the seasonal density of blue sheep (Liu et al., 2008), analyzing the effect of food on the distribution of blue sheep (Namgail et al., 2009) and assessing the dietary structure of blue sheep (Aryal et al., 2015). Few studies on how the hooves of blue sheep achieve their biomechanical functions during locomotion have been reported. To the best of our knowledge, only a few studies have analyzed the biomechanical functions of goat hooves, but goats have weaker climbing abilities than blue sheep. Most previous studies (Carroll et al., 2008; Lee et al., 2008; Arnold et al., 2013) analyzed the goat hoof as a whole to discuss the moving characteristics because hooves were thought to contribute little to forward locomotion (Fischer and Blickhan, 2006); however, this generalization may underestimate the importance of the hoof. Some further investigations proposed that the cloven hooves of goats may spread apart and actively “grasp” the rock to prevent slipping during locomotion (Manning et al., 1990). Cloven hooves could adjust attitude by changing the relative positions between the two digits in the swing phase; this function helps achieve a better landing orientation. In addition, goats can adjust the position of the metacarpophalangeal joint or metatarsophalangeal joint (MTP or MCP joints) with no relative motion between the hoof tip and the ground, which also ensures strong ground contact and dexterity in the stance phase (Zhang et al., 2015). Zhang et al. (2017) modeled goat hooves as an equivalent mechanism with decoupled flexion-extension and lateral movement. The upper part of the equivalent mechanism can flex and extend, while the lower part performs the lateral movement. By combining these two parts, the mechanism can adapt to the longitudinal slope (anterior-posterior) and transverse slope (medial-lateral). Based on this model, they explained that the mechanism ensures terrain adaptability by selecting the appropriate configuration for the terrain conditions (Zhang et al., 2017). Similarly, Abad et al. (2016) proposed a multibody compliant robotic foot inspired by goat hooves and presented analytical and experimental explanations for how the morphological computations performed by the goat hoof significantly reduce slipping on both smooth and rough surfaces. A recent experimental study of goat hooves found that pore structures with inclination angles of 55° existed in the hoof capsule and contributed by cushioning impacts (Tian et al., 2019).

Although previous studies have greatly improved our understanding of the functions of goat and blue sheep hooves due to the similarities in the hooves of these two mountain ungulates, little is known about the microstructure, mechanical properties, or surface morphology of blue sheep hooves and the potential effects of these parameters related to biological functions such as contact stability, which may provide critical information for the development of novel biomimetic robot feet with enhanced properties such as anti-slip, impact cushioning and impact resistance.

The objective of this study was to investigate the microstructure, surface morphology and mechanical properties of a blue sheep hoof using field-emission scanning electron microscopy (FESEM), energy-dispersive X-ray spectroscopy (EDS), Fourier transform infrared (FTIR) spectroscopy, nanoindentation and finite element (FE) simulations and to correlate these structural and material properties to the passive contact stability function of the hoof.

## Materials and Methods

### Ethics Statement

This study was approved by the Institutional Review Board Committee of Jilin University, Changchun, China (No. 20170925).

### Preparation of the Blue Sheep Hoof

Blue sheep are under second-class national protection in China, and both the hunting and trading of blue sheep are prohibited. Hooves of a dead blue sheep were donated by a local botanical garden strictly for scientific research. The sheep was 4 years old and had no musculoskeletal disease. The stratum corneum of hooves were fully formed. The left front hoof and left hind hoof of the blue sheep were removed with a scalpel and soaked in a formalin solution for preservation.

### Microstructure, Material Composition, and Surface Morphology Characterization

**FIGURE 1:**
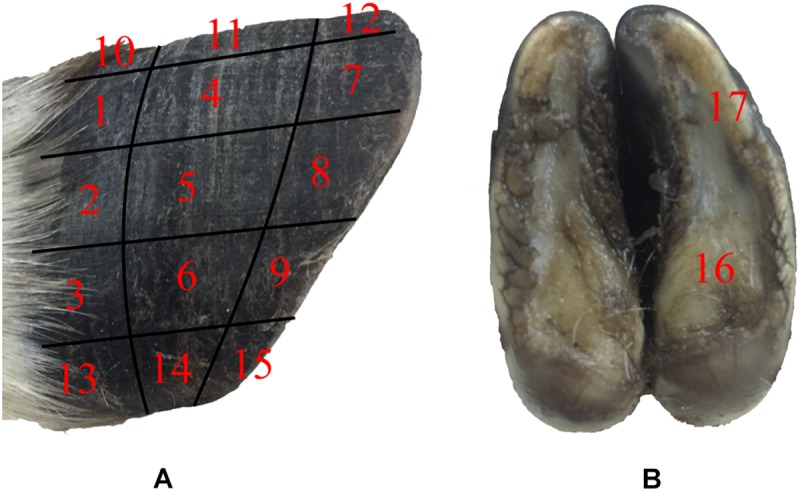
Sampling locations for stereomicroscope observations and friction coefficient measurements. **(A)** lateral surfaces of hoof, **(B)** hoof bottom.

The surface morphology of the blue sheep hoof was characterized with a stereomicroscope (Stemi 508, ZEISS, Germany). The lateral surfaces of the front and rear hooves were observed, and the observed areas of these hooves were divided into 15 regions, as shown in Figure 1A.

**FIGURE 2:**
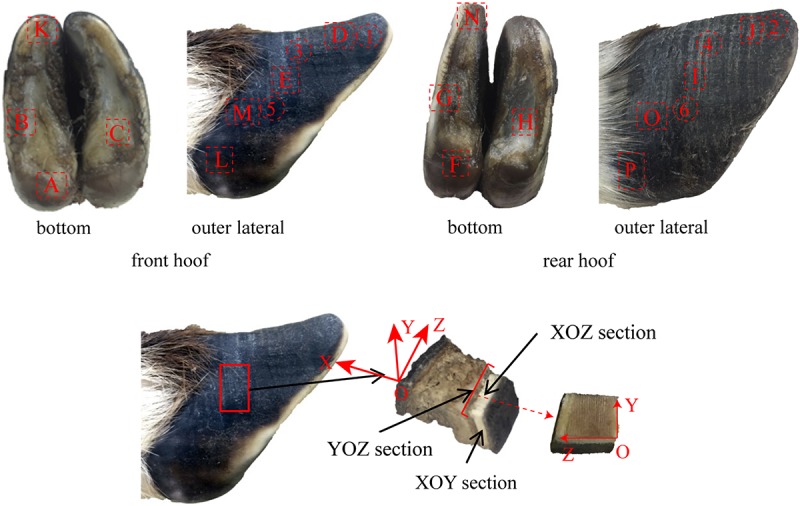
Sampling locations for cross-sectional microstructure and surface morphology observations.

The microstructure, material composition and surface morphology of the blue sheep hooves were characterized using FESEM (XL-30, FEI Company, United States), which is equipped with EDS (Genesis 2000, EDAX Company). Separate samples for surface morphology and for cross-sectional microstructure observation were cut from the hooves. The samples used for surface morphology observations were taken from the blue sheep’s front and rear hooves. The samples cut from the front hoof were taken from regions B, K, D, E, M, and L, and the samples cut from the hind hoof were taken from regions G, N, J, I, O, and P, as shown in Figure 2. Each sample was ∼6 mm × 5 mm × 5 mm (length × width × thickness). For cross-sectional observations, the samples were obtained by fracturing the hoof immediately before examination.

The samples were ultrasonically cleaned in distilled water for 20 min, and then the samples were placed in a −86°C ultra-low freezer (DW-HL508, Zhongke Meiling Cryogenic Technology Co., Ltd., Hefei, China) for 6 h and dried in a freeze drier (FD-1C-50, Beijing Boyikang Experimental Instrument Co., Ltd., Beijing, China) for 24 h. All samples were mounted on aluminum sample holders and coated with 10 nm of gold in a sputter coater (JFC-1600, JEOL Ltd.). The samples were measured in secondary electron mode at accelerating voltages of 10 and 20 kV. In addition, the material composition of each sample was analyzed by the EDS module of the field-emission scanning electron microscope.

The surface distribution of elements in the cross section of the stratum corneum was analyzed with an energy-dispersive spectrometer (X-MAX OXFORD INSTRUMENTS). The six samples were obtained from regions 1–6 (see Figure 2). The YOZ plane of each sample was ground flat prior to performing EDS analysis of the sample.

An FTIR spectrometer (IRAffinity-1, Shimadzu, Japan) was used to collect the infrared spectra of the stratum corneum from the blue sheep hoof to analyze the main chemical groups present. The highest resolution of the instrument is 0.5 cm^–1^, and the scanned wavelength range was 7,800–350 cm^–1^. The FTIR spectra were acquired in attenuated total reflectance (ATR) mode. Attenuated total reflectance (ATR) mid-infrared (MIR) spectroscopy is a non-invasive technique for functional group analysis (Kamal and Karoui, 2015; Wu et al., 2015; Hell et al., 2016). This technique has the advantages of high speed, not requiring reagents and being non-destructive (Karoui et al., 2010).

Ten samples were taken from regions A–J of the front and rear hooves of the blue sheep, as shown in Figure 2. The samples were ultrasonically cleaned in distilled water for 20 min, and then the samples were placed in a −86°C ultra-low freezer (DW-HL508, Zhongke Meiling Cryogenic Technology Co., Ltd., Hefei, China) for 6 h and dried in a freeze drier (FD-1C-50, Beijing Boyikang Experimental Instrument Co., Ltd., Beijing, China) for 24 h. KBr (200 mg) was ground into a powder to prepare a background tablet. Two milligrams of the powdered, dried hoof cross section was ground with 200 mg of KBr and pressed to form samples for analysis. A distinct 4-layer structure was observed in the cross section of the stratum corneum of the hoof, and a pressed sample was prepared for each layer to analyze the chemical groups present in the different layers. The infrared spectra of the background tablet and the sample were measured by a FTIR spectrometer. The infrared spectra were prepared and analyzed by MATLAB software (R2014a, MathWorks).

### Mechanical Property Measurements

**FIGURE 3:**
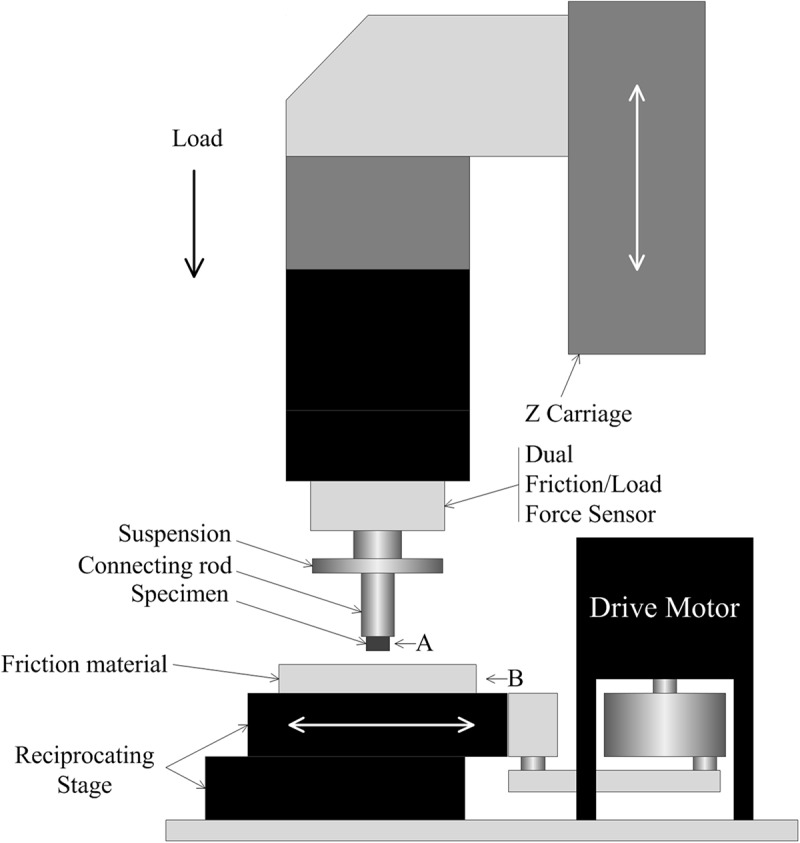
Diagram of the measurements of the friction coefficients. **(A)** specimen installation location, **(B)** friction material installation location.

The tribological properties of the surface of blue sheep hooves at a microscopic level were experimentally determined. The friction coefficients at different positions on the surface of the blue sheep hooves were measured by a Nano Tribometer (UNMT-1, CETR Company, United States). During the experiment, the sample is placed at position A, and the friction material is placed at position B (see Figure 3). The motion of mode used to assess friction was reciprocating motion, the motion speed was 1 mm/s, the motion distance was 5 mm, the applied load was −2 N, and the friction material was steel. The sample size is 4 mm × 6 mm (width × length) strip, and the length was used as the direction of sliding. The bottom of the sample was ground with sandpaper, glued to an iron column and fixed to the connecting rod. Fifteen samples were taken from the lateral side of the stratum corneum of the anterior hoof at the same sampling locations as those observed by stereomicroscope (see Figure 1A). The other two samples were taken from the lateral side and center region of the hoof bottom, as shown in Figure 1B.

Unlike traditional uniaxial measurements, which provide information only on overall mechanical properties, nanoindentation can provide site-specific data regarding the modulus and hardness of a sample at the nano- or microscale. Therefore, to study the differences in mechanical properties between different layers of the blue sheep’s hoof, nanoindentation tests were conducted on the cross section of each layer from the outside to the inside.

One sample was prepared for the nanoindentation test. The sample was taken from region E, as shown in Figure 2. A single point was tested for each layer from the outermost layer to the innermost layer, i.e., there were four points tested in total. Ten indentations were performed at each point. The samples were used to analyze the variations in hardness and modulus from the outermost layer to the innermost layer. The experimental procedure for assessing indentation adopts load feedback as the control mode. The loading time was 5 s, the holding time was 2 s, and the unloading time was 5 s. The load was linearly increased from 500 to 1,500 μN. Prior to nanoindentation testing, each sample was finely polished (MP-2, Shanghai Aidu Energy Technology Co., Ltd.) (Chen et al., 2008b). All samples were cleaned for 30 min in an ultrasonic cleaner (KQ-50DA, Kunshan ultrasonic instruments Co., Ltd.) and then dried at room temperature. A Keysight nanoindenter (G200, Chandler, AZ, United States) was used for the nanoindentation tests. A three-sided diamond pyramidal Berkovich tip was used for all experiments. The indentation data were analyzed with the Oliver and Pharr method (Oliver and Pharr, 2004; Shu et al., 2007).

### Finite Element Simulation

The observed morphology is described in detail in the results section (Surface Morphology). However, to clearly describe the FE simulation, it must be noted that straight and curled lamellar morphologies (see Figure 6a) were observed on the surface of the hoof, and four-layer structures (see Figure 7e) were observed in the cross section of the hoof. The purpose of the FE simulation was to investigate both the effect of the lamellar morphology on increasing surface friction and the effect of the four-layer structure on contact cushioning.

**FIGURE 4:**
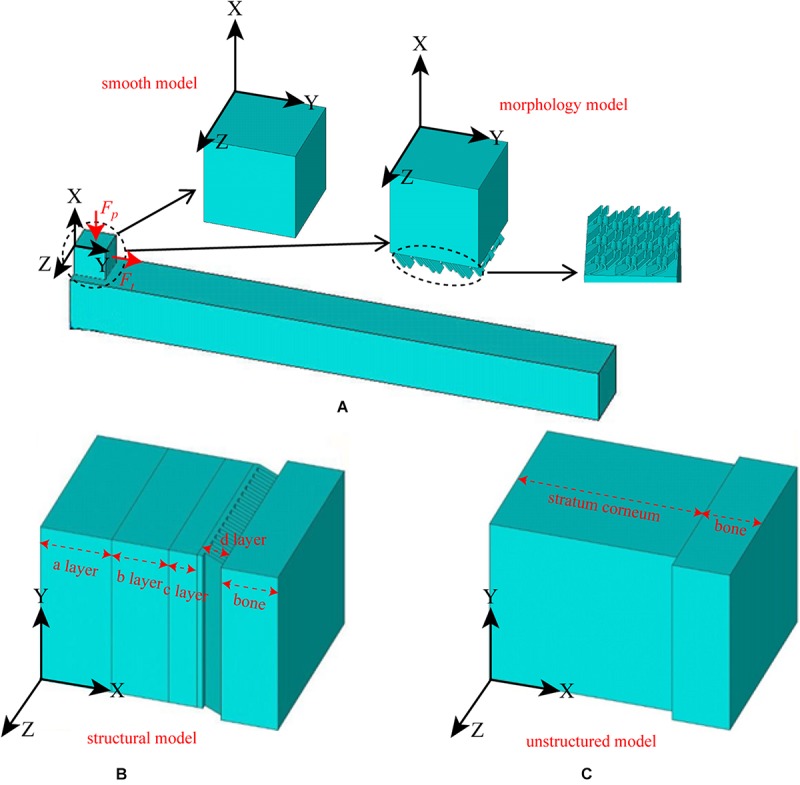
Constructed FE models. **(A)** morphological model and its contrast model, **(B)** structural model, and **(C)** contrast model of the structural model.

Therefore, two types of models, namely, the morphology model and structural model, and their contrast models were constructed. The surface of the morphology model had both straight and curled lamellar morphological features, whereas the contrast model had a smooth surface (see Figure 4A). The structural model (see Figure 4B) had two forms: one was structural model A, which had a four-layer structure, and each layer had different material properties; the other was structural model B, which had the same structure as structural model A, but each layer had the same material properties. The contrast model of the structural mode was an unstructured model with no hierarchical structure and homogeneous material properties (see Figure 4C). CATIA (V5R21, Dassault Aviation, Paris, France) was used to build the geometric model of each model, and then the models were imported into the FE software package ANSYS (V16.0, ANSYS, Pittsburgh, United States).

A 3D solid beam was used to simulate the ground with its bottom surface fully fixed. The interaction between the hoof side surface and the ground was defined to have a frictional coefficient of 0.6. The horizontal direction allowed arbitrary separation, sliding and rotation of the contact surfaces (Akrami et al., 2018). The material properties of each part were all idealized as being homogenous isotropic and linear elastic with different Young’s moduli and Poisson’s ratios. The material properties and element types used for the morphology model, the smooth model and the ground model are listed in Table 1. The mesh was determined through a convergence analysis by gradually increasing the mesh density until the deviations in the estimated stresses reached <5%. The sum of the mesh numbers of the morphology model and ground model is 501785, whereas the sum of the mesh numbers of the smooth model and ground model is 79254. Uniformly distributed pressure (Fp) was applied to the upper surface of the model, and tension (Ft) was applied along the Y-axis. A preconditioned solver (PCG) was used, and the time step size was 0.02 s. The structural model components from the outside to the inside are layer a, layer b, layer c, layer d and bone, whereas the unstructured model components are stratum corneum and bone. The material properties of structural model A, structural model B and the unstructured model were idealized being homogenous isotropic and linear elastic with different Young’s moduli and Poisson’s ratios. The material properties and element types used for modeling different components of the stratum corneum and bone are listed in Table 2. A displacement constraint was applied along the Y-axis to prevent the elements from shifting to the left along the Y-axis; this displacement constraint simulated a rock wall. A velocity of −2 m/s was applied to the structural model and the unstructured model along the Y-axis. The mesh density was determined by convergence analysis, and the mesh numbers of the structural model and the unstructured model were 39,150 and 40,500, respectively. The full transient analysis method was used, the solver was a sparse matrix solver, and the loading method was stepped loading. The total time was defined as 0.01 s.

**TABLE 1:** Material properties and element types of the FE models for friction analysis.

**Model**	**Material**	**Element type**	**Young’s modulus (MPa)**	**Poisson’s ratio**	**References**
Morphology model	Solid, linear elastic	Tetrahedral	4,500	0.38	Yingjie, 2005
Smooth model	Solid, linear elastic	Tetrahedral	4,500	0.38	Yingjie, 2005
Ground model	Solid, linear elastic	Tetrahedral	17,000	0.1	Akrami et al., 2018
					

**TABLE 2:** Material properties and element types of the FE models for impact cushioning analysis.

**Model**	**Component**	**Material**	**Element type**	**Young’s modulus (MPa)**	**Poisson’s ratio**	**References**
Structural model A	Bone	Solid, linear elastic	Hexahedral	7,300	0.3	Dai et al., 2006
	Layer a	Solid, linear elastic	Hexahedral	4,500	0.38	Yingjie, 2005
	Layer b	Solid, linear elastic	Hexahedral	3,880	0.38	Yingjie, 2005
	Layer c	Solid, linear elastic	Hexahedral	4,170	0.38	Yingjie, 2005
	Layer d	Solid, linear elastic	Hexahedral	4,010	0.38	Yingjie, 2005
Structural model B	Bone	Solid, linear elastic	Hexahedral	7,300	0.3	Dai et al., 2006
	Layers a–d	Solid, linear elastic	Hexahedral	4,500	0.38	Yingjie, 2005
Unstructured model	Bone	Solid, linear elastic	Hexahedral	7,300	0.3	Dai et al., 2006
	Stratum corneum	Solid, linear elastic	Hexahedral	4,500	0.38	Yingjie, 2005

### Statistical Analysis

Statistically significant differences in hardness and modulus strength among the different layers and friction coefficients of different regions were identified by using one-way analysis of variance (ANOVA). The statistical significance level for the ANOVA was assumed to be 0.05.

## Results

### Surface Morphology

**FIGURE 5:**
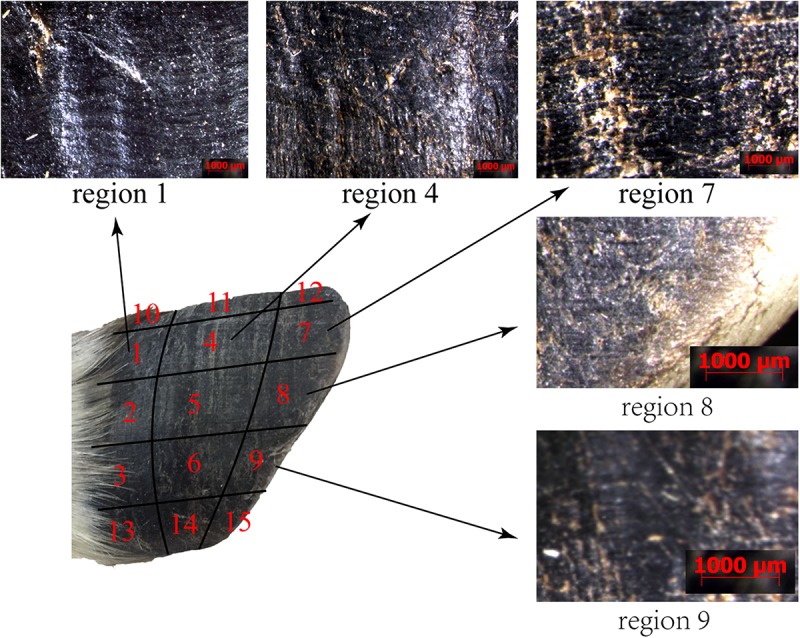
Stereomicroscopy micrographs of the surface morphologies of different regions.

Figure 5 shows that regions 1, 4, 7, 8, and 9 of the front and rear hooves have longitudinal stripes. The longitudinal stripes of region 7 have a width of 137.23 ± 16.68 μm and a stripe spacing of 233.71 ± 27.81 μm. The scale of the longitudinal stripes gradually decreases from region 7 along the longitudinal direction to region 1 and in the lateral direction to region 9. No stripe morphology was found in other regions.

**FIGURE 6:**
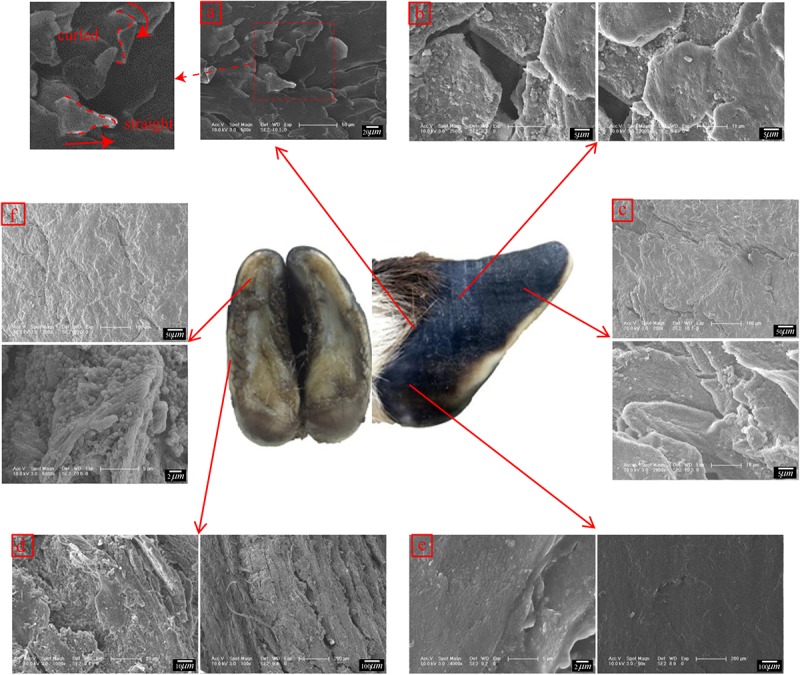
Scanning electron micrographs of the surface morphologies of different regions. **(a)** surface morphology of region M, **(b)** surface morphology of region E, **(c)** surface morphology of region D, **(d)** surface morphology of region B, **(e)** surface morphology of region L, and **(f)** surface morphology of region K.

Figure 6a shows that straight and curled lamellar morphological features were distributed in region M. Figure 6b shows that region E was superimposed with irregular polygons with side lengths of 15.77 ± 1.96 μm. There was a crack in the longitudinal direction in the sample taken from the hoof tip (region D) in Figure 6c. A fusiform plate-like structure was superimposed near the crack.

Figure 6d shows that region B was superimposed with a vertical plate-like structure. Figure 6e shows that region L was smooth and without obvious delamination or block structures. There were irregular cracks in region K, as shown in Figure 6f. Within the cracks, there was a plate structure with a surface-distributed stripe, and a layer of spherical particles was present between adjacent plates.

### Structural Characterization of the Cross Section

**FIGURE 7:**
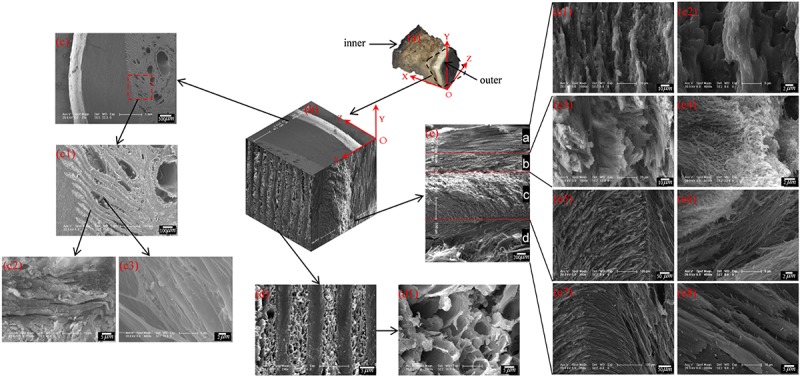
Scanning electron micrographs of a cross section of the blue sheep hoof at different magnifications. **(a)** position of the sample and the coordinate system established, **(b)** 3D view of stratum corneum section of blue sheep hoof, **(c)** sectional structure of the XOZ surface of the sheep’s hoof capsule, **(d)** sectional structure of the YOZ surface of the sheep’s hoof capsule, and **(e)** sectional structure of the XOY surface of the sheep’s hoof capsule.

To describe the cross-sectional structure, a coordinate system is established (see Figure 7a). The XOY section of the sample cut from region E shows that the stratum corneum is a four-layer structure comprising layer a, layer b, layer c and layer d, as shown in Figure 7e. The thicknesses of layers a, b, c, and d are 450–665 μm, 390–460 μm, 1,066–1,200 μm, and 533–666 μm, respectively. Figure 7e1 shows that layer a is a dense lamellar layer, and the thickness of the lamellar morphology is 6.67 μm. Figure 7e3 shows that layer b is composed of crossed fiber bundles, and the fiber bundles are 5.12 μm thick. Figures 7e5–e8 shows that layers c and d are composed of keratin fiber bundles. Layer c is denser than layer d, and the directions of the fiber bundles in layers c and d are approximately symmetrically distributed around the interface of the two layers.

Figure 7d shows the cross-sectional structure of the YOZ surface of the sheep’s hoof capsule. Layer d is composed of plate layer structures and porous structures, and the plate layer structures (Figure 7c1) are composed of several lamellar morphologies (Figure 7c2) with several vertical fiber combinations (Figure 7c3). The porous structure is composed of pore with diameters of 2.5–13.5 μm. Figure 7b presents a three-dimensional diagram created by electron microscopy of the front, top and left side sections of the hoof, showing the three-dimensional structure of the blue sheep’s hoof capsule.

### Composition Analysis

#### Elemental Mapping Analysis of the Cross Sections

**FIGURE 8:**
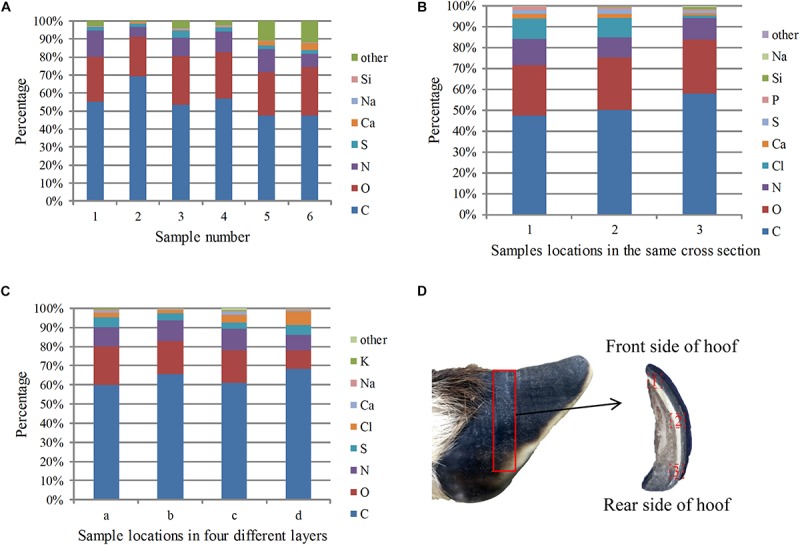
EDS analysis results of the cross sections of the samples: **(A)** percentage of each element in the different sections, **(B)** percentage of each element at different locations in the same cross section from the front side to the rear side, **(C)** percentage of elements in the four layers from the outside to the inside, and **(D)** sampling locations for the EDS analysis in different areas of the same plane.

When SEM was performed, each element was synchronously mapped by EDS. As shown in Figure 8A, the three most abundant elements in the different samples were C, O, and N, and their proportions were 54.98, 25.08, and 10.30%, respectively. Other minor elements, including Mg, P, Cl, K, and Fe, were also present in the samples.

To study the distribution of elements in the stratum corneum from front to back, EDS analysis was carried out on a cross section from the middle of the stratum corneum. The sampling location is shown in Figure 8D, and the results are shown in Figure 8B. The total carbon, oxygen and nitrogen contents increased gradually from front to back, indicating that the amide and keratin contents increased. The calcium content decreased, which may indicate that the degree of calcification decreased from front to back.

Figure 8C shows the relative content of each element from the outermost to the innermost of the four layers. The figure shows that carbon, oxygen and nitrogen were uniformly distributed, whereas the sulfur content gradually reduced from the outer layer to the inner layer. Sulfur was mainly present in the proteins in the hoof nail, and the decreased sulfur content indicates that the protein content decreased between sequential layers in the stratum corneum. The content of calcium was higher in the outermost layer than in the second layer, indicating that the outermost layer has a higher degree of mineralization.

#### Infrared Spectrum Analysis

**FIGURE 9:**
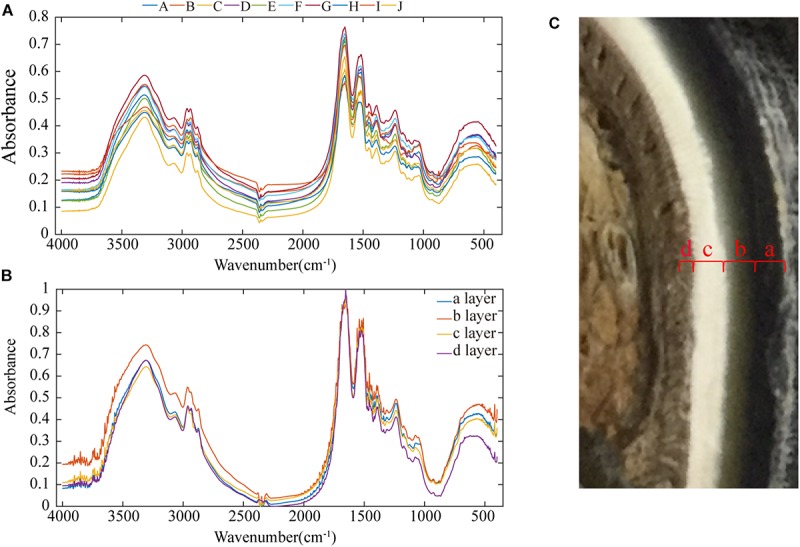
Infrared spectra of the samples: **(A)** infrared spectra of all samples, **(B)** infrared spectra of each of the four layers of the stratum corneum, and **(C)** cross-sectional image showing that the stratum corneum is divided into four layers.

Figure 9A shows the infrared spectra of all the samples, and the spectra of all the samples have the same peaks. The signals in the fingerprint region result from a multitude of bending vibrations in complex molecules. The fingerprint signals of each compound are unique, and these signals can therefore be taken as strong evidence to confirm whether two spectra are identical. Figure 9A shows that the fingerprint regions of all the spectra are very similar, so the functional groups present in all the samples are basically the same.

In Figure 9A, a broad absorption peak exists at ∼3,400 cm^–1^, and this peak is broadened by the superposition of the −OH stretching vibration absorption peak of groups involved in hydrogen bonding and the stretching vibration absorption peak of −NH. Two absorption bands for the stretching vibrations of C–H exist at ∼2,918 and 2,880 cm^–1^. There are two absorption peaks at ∼2,961 and 2,874 cm^–1^, and these correspond to the anti-symmetric CH stretching vibration and the symmetric CH stretching vibration of −CH_3_ groups. The absorption peak at ∼1,655 cm^–1^ is the amide I CO stretching vibration, the absorption peak at 1,520–1,541 cm^–1^ is the amide II CN stretching vibration and NH in-plane deformation vibration, and the absorption peak at ∼1,238 cm^–1^ is the amide III CN stretching vibration and NH in-plane deformation vibration. The three amide absorption bands in these spectra indicate that the secondary amides in the keratin in these samples are all involved in hydrogen bonding. The absorption peak at ∼1,387 cm^–1^ is the C–H bending vibration and −CH_3_ symmetric deformation vibration, and the absorption peak at ∼1456 cm^–1^ is the CH_2_ bending vibration and −CH_3_ deformation vibration.

Figure 9C shows that the inner part of the stratum corneum is clearly divided into four layers, and each layer was subjected to separate infrared spectral analyses (see Figure 9B). The size of the peak area indicates the content of the functional group. Layer b has more intense –S–C peaks (1051 cm^–1^) relative to those of the other three layers, showing a cysteine-rich composition (Zhang et al., 2008). The presence of a cystine monoxide (S = O) peak (1076 cm^–1^) indicates the oxidation of cystine, which could be due to the influence of the stratum corneum preservation method (Chou et al., 2012; Fresnais et al., 2015). According to the number of disulfide bonds in cystine, keratin materials can be classified as soft or hard keratin (Zhang et al., 2018).

### Mechanical Properties

**FIGURE 10:**
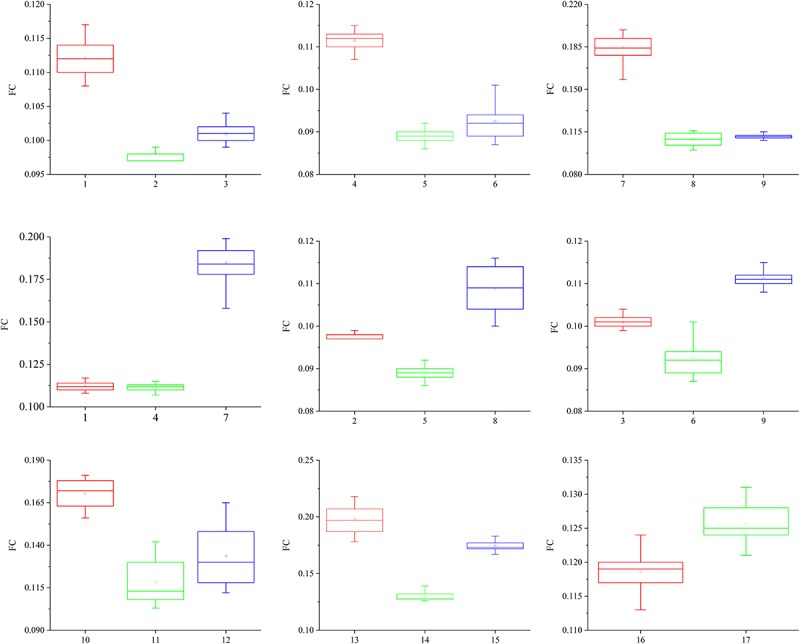
Contrast diagram of the FCs of the different regions of the stratum corneum.

The friction coefficients (FCs) of all the samples are shown in Table 3 and Figure 10. The FCs of the samples were obviously different (*P* = 0.005), as shown in Figure 10. The trends in the FCs among each position of the stratum corneum in the longitudinal direction are 7 > 1 > 4, 8 > 2 > 5, 9 > 3 > 6, and 10 > 12 > 11, and those in the horizontal direction are 1 > 3 > 2, 4 > 6 > 5, and 7 > 9 > 8. As shown in Figure 10, the FCs on the lateral side of the hoof bottom are greater than those of the center region of the hoof bottom.

**TABLE 3:** FC in different regions of the stratum corneum.

**Region**	**FC**	**Region**	**FC**	**Region**	**FC**
1	0.1123 ± 0.0026	2	0.0979 ± 0.0007	3	0.1010 ± 0.0013
4	0.1115 ± 0.0023	5	0.0893 ± 0.0014	6	0.0925 ± 0.0044
7	0.1847 ± 0.0089	8	0.1088 ± 0.0052	9	0.1112 ± 0.0018
10	0.1704 ± 0.0082	11	0.1182 ± 0.0124	12	0.1339 ± 0.1339
13	0.1979 ± 0.0039	14	0.1301 ± 0.0039	15	0.1739 ± 0.0040
16	0.1186 ± 0.0030	17	0.1257 ± 0.0025		

**FIGURE 11:**
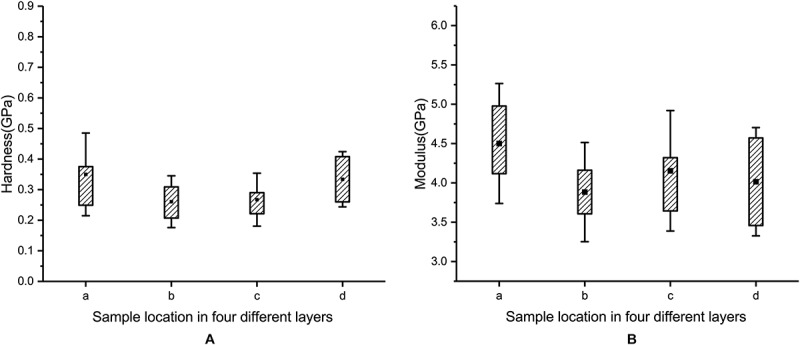
Measured hardness **(A)** and modulus **(B)** for each of the four layers.

The hardness values of layer a, layer b, layer c, and layer d are 0.35 ± 0.14 GPa, 0.26 ± 0.08 GPa, 0.27 ± 0.09 GPa, and 0.33 ± 0.09 GPa, respectively (see Figure 11A). The moduli of layer a, layer b, layer c, and layer d are 4.50 ± 0.76 GPa, 3.88 ± 0.63 GPa, 4.17 ± 0.78 GPa, and 4.01 ± 0.69 GPa, respectively (see Figure 11B). A comparison of the results shows that the hardness and modulus of layer a are 35 and 16% higher than those of layer b, respectively. Therefore, the outermost layer of the blue sheep’s hooves is stronger than the inner three layers and more resistant to external loads.

### Finite Element Simulation Analysis

According to Newton’s second law, *F* = *ma*, where *F* = *F*_*t*_ *−f*. Moreover, the displacement is *S* = 1/2 *at*^2^. Thus,

$$S=\frac{1}{2}\,\frac{F_{t}-f}{m}\,t^{2}$$

where *F*_*t*_ is the tension applied along the Y-axis, *f* is the friction between the hoof side surface and the ground, *S* is the displacement of the smooth model and the bionic model, and *t* is the simulation time. The displacement, *S*, is negatively correlated to the frictional force, *f*.

**FIGURE 12:**
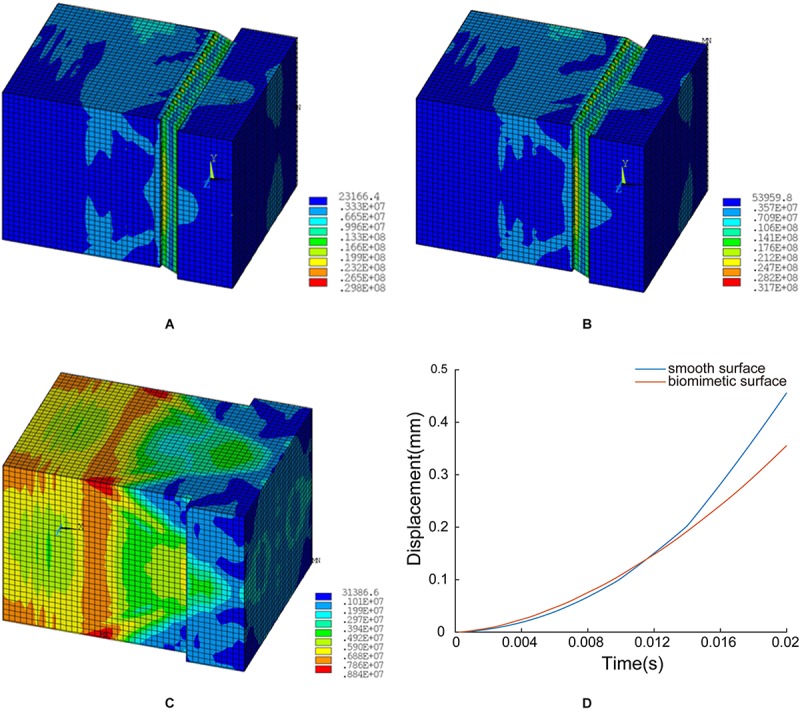
FE simulation results: **(A)** structural model A, **(B)** structural model B, **(C)** unstructured model, and **(D)** time-displacement graphs of the two models.

Figure 12D shows the displacement time plots of the smooth model and the bionic model. The displacements of the smooth model and the bionic model at 0.02 s are 4.56 × 10^–1^ mm and 3.56 × 10^–1^ mm, respectively, which confirms that the surface morphology contributes to the anti-slip function and improves the contact stability of the blue sheep hoof.

In structural model A, the maximum von Mises stress is 29.8 MPa, and the minimum value is 0.23 MPa. The stress is mainly concentrated at the ends of layer d (inclined layer), and the maximum stress on the innermost stressed bone is 0.53 MPa (see Figure 12A).

In structural model B, the maximum stress is 31.7 MPa, and the minimum value is 0.54 MPa. The stress is primarily concentrated at the ends of layer d (inclined layer), and the maximum stress on the innermost stressed bone is 0.62 MPa (see Figure 12B).

In the unstructured model, the maximum stress is 8.8 MPa, and the minimum value is 0.31 MPa. The stress is predominantly concentrated at the corresponding position of layer c in the biological model, and the maximum stress on the innermost stressed bone is 0.59 MPa (see Figure 12C).

## Discussion

Previous studies have found that blue sheep have excellent climbing abilities. During climbing, the hoof is the only part to contact the ground and rock. Therefore, the hoof of a blue sheep must offer both impact resistance and slip resistance to ensure stable contact between the hoof and the ground or rock. This study experimentally characterized the microstructure, surface morphology, material composition and mechanical properties of blue sheep hooves to analyze the potential contributions of these properties to establishing passive contact stability. The stereomicroscope results show that regions 1, 4, 7, 8, and 9 of the front and rear hooves have longitudinal stripes. Combined with the measured FCs, these results suggest that the FCs of the areas with stripes are significantly higher than those of the areas without stripes in both the transverse direction and longitudinal direction. Therefore, the stripe morphology on the surface of the stratum corneum of the blue sheep hooves could contribute to the anti-slipping ability of the hoof. Microscopic straight and curled lamellar morphological features were also observed on the surface of the blue sheep’s hooves, and such features had not been reported in previous studies of goat hooves. These lamellar morphologies may affect the interactions between the hooves and rocks. The surface morphology helps increase the friction between the hoof and the rock in a manner similar to the spines on a desert locust’s foot, which increases friction by penetrating the ground or the hollow of the rock. Previous experimental and modeling investigations have shown that a desert locust reduces slippage by adjusting the angle of the spines and using the spines to puncture the concave surface of the ground or rock surface (Woodward and Sitti, 2018). The experimentally determined FCs showed that in the same longitudinal direction, the FCs of samples 1, 2, and 3 taken from regions with a lamellar morphology are larger than those of samples taken from the corresponding regions without lamellar morphologies (samples 4, 5, and 6, respectively). To further verify the possible anti-slip function of the barbed structures on the surface of the sheep’s hooves, FE simulations with a smooth model and a bionic model were conducted in this study. The FE simulation results suggest that the straight and curled lamellar morphologies play a role in the climbing ability of blue sheep by increasing the friction between the blue sheep’s hoof and the rock or ground. Our analysis showed that the surface of the hooves of the blue sheep increases the friction force when in contact with rock/ground through a combination of macroscopic striation morphology and microscopic lamellar morphology, which will enhance stability, skid resistance and contact as the sheep climbs. However, only the microscopic lamellar morphology was analyzed in this study, and future studies must investigate the effects of the whole hierarchical morphology on hoof function.

The microstructures of cross sections of hooves were obtained in the study. The stratum corneum of the sheep’s hooves is a four-layer structure, wherein layer a (the outermost layer) consists of many densely stacked sublayers, layer b consists of crossed fiber bundles, and layers c and d are composed of fiber bundles that are symmetrically distributed. To the best of our knowledge, this structure has not been reported in the stratum corneum of the hooves of other artiodactyls. Stratified structures have been found in equine hooves (Kasapi and Gosline, 1999) and sheep horns (Tombolato et al., 2010). Previous experiments have shown that layered structures in horns can enhance toughness (Kitchener, 1987). Delamination is evident in our samples in the regions of layer a and layer b. Similar crossed fiber bundles have been found in the skeletons of arthropods and the carapaces of crustaceans (Raabe et al., 2005; Chen et al., 2008b; Weaver et al., 2012; Chandran et al., 2016; Qian et al., 2018). Previous studies have shown that this cross-helicoidal structure can support an animal’s body weight, enhance toughness and increase impact resistance (Chen et al., 2008b, 2014; Yang et al., 2017). Therefore, the microstructure of the stratum corneum provides excellent mechanical properties and is resistant to damage when the blue sheep’s hooves collide with rock.

The infrared spectra showed that the four-layer structure of the sheep’s hooves contained keratin, which has been widely found in the wool, hair, hooves and horns of mammals as well as bird feathers, beaks and reptilian claws. Previous studies have shown that keratin has remarkable mechanical efficiency because of its relatively high fracture toughness and Young’s modulus, and is much lighter than other highly mineralized biological materials, such as bone and teeth (Wegst and Ashby, 2004; Meyers et al., 2008). Therefore, the presence of keratin can increase the impact resistance and fracture toughness of the stratum corneum of blue sheep’s hooves and protect the internal tissues from injury for a long time. EDS analysis showed that the samples contained carbon, oxygen, nitrogen, sulfur and calcium, which was expected; in particular, the sulfur content suggests the presence of cysteine (Tombolato et al., 2010). α-Keratin is a structural fibrous protein that exists in wool, hair, mammalian hooves, and horns. The keratin molecules are held together by H-bonding and disulfide cross-links due to the presence of cysteine. The disulfide bridges make the structure quite rigid (Tombolato et al., 2010). The change in the calcium content indicates that the degree of calcification of layer a is higher than that of layer b, and this is reminiscent of the exoskeletons of crayfish and crabs, in which the mechanical rigidity is increased by a high degree of calcification (Chen et al., 2008b). This structure offers a high surface hardness and good wear resistance, and these structures widely exist in nature. For instance, the outermost layer of the shell of American lobsters is more mineralized than the inner epidermis (Raabe et al., 2006). Another example is mammalian teeth, which are composed of external enamel and internal dentin; enamel is solid and highly mineralized, whereas dentin is hard and contains 30% collagen (Chen et al., 2008b).

Similarly, both blue sheep hooves and goat hooves have hard stratum corneum on the sides and soft tissue in the center of the bottom. But the tratum corneum surface of blue sheep hoof has lamellar and striated morphology, which has not been reported for goat so far. Meantime, layered structures were found in cross section of blue sheep hoof, while previous studies showed the goat hoof has inclined tubule structure inside (Tian et al., 2019). Those different morphology and structures of hoof may help explain why blue sheep has advantageous over goat during climbing of cliff or irregular terrains.

Nanoindentation measurements of the different layers in the cross section of the hoof show that both the modulus and hardness exhibit a high-low-high-low distribution trend from layer a to layer d. Similarly, the modulus and hardness in sheep hooves, crab claws, snapping shrimp claws and keratinous cattle horns also exhibit gradient variations (Chen et al., 2008a; Sun et al., 2012; Qian et al., 2018). Layer a has a lamellar structure and is stacked more densely than layer b, as shown in Figure 4. The modulus and hardness gradients of layers a and b may arise from the gradations in different fiber orientations and the stacking density (Lian and Wang, 2014). The modulus and hardness in crab claws (Lian and Wang, 2014) and American lobster claws (Sun et al., 2012) are also discontinuous. This design (high surface hardness and surface wear resistance) is common in nature (Lian and Wang, 2014). This gradient modulus can effectively suppress microcracks at the interfaces when subjected to external loads (Suresh, 2001).

A recent study reported that the ratio between the indentation hardness (*H*_*I*_) and modulus (*E*_*I*_) of a biological material is a key parameter that serves as a proxy for the ratio between irreversible and reversible deformation in the contact zone during indentation and the material’s yield strength (Labonte et al., 2017), which facilitates the quantitative comparison and evaluation of structural biological materials. In this work (Labonte et al., 2017), the elastic index (*I*_*E*_) was determined as follows:

$$I_{E}=H_{I}/E_{I}$$

Moreover, the yield strength (σ_Y_) of the material was obtained by the following equations:

$$\sigma_{\text{Y}}=H_{I}/\gamma$$

$$\gamma =\frac{2\,A_{1}}{\tan\left(\beta \right)}\,I_{B}\,\frac{1}{\alpha \tan h(2/\tan\left(\beta \right)I_{E}}$$

where *A*_1_ is a constant depending on the indenter geometry and the yield criterion (for a Berkovich tip and the von Mises yield criterion, *A*_1_≈ 2.75; β = 19.7; Yu and Blanchard, 1996; Labonte et al., 2017). The elastic index and yield strength of layer a, layer b, layer c, and layer d were calculated based on the modulus and hardness data. The values of the elastic index, *I*_*E*_, from layer a to layer d are 0.078, 0.067, 0.065, and 0.082. All of the obtained *I*_*E*_ values are within the range of 0.01–0.1, which is the range previously determined for biological materials (Labonte et al., 2017). The yield strengths from layer a to layer d are 2.567 GPa, 2.616 GPa, 2.626 GPa, and 2.544 GPa.

To verify the possible impact resistance function of the layered structure and gradient modulus, FE simulations of the biological model and the comparison model were conducted in this study.

The FE simulation results suggested that the hard-soft coupling of structural model A can reduce the stress transmitted to the bone during impact (see Figures 8A,B). Therefore, the hard-soft coupling structure of the blue sheep’s hoof contributes to the dissipation of the instantaneous ground force applied to the hoof stratum corneum, thereby increasing the contact stability.

Structural model B can reduce the stress on the internal skeleton and change the distribution of stress (as shown in Figures 8B,C). This structural model can reduce the stress on the innermost bone. In addition, the distribution of stress is changed so that the stress protrudes inward in the stratum corneum, which can prevent damage due to the uneven distribution of stress on the bone. Therefore, our results may confirm that the innermost inclined slab structure of the blue sheep hoof can reduce the stress on the bone, change the distribution of stress during impact, and prevent deformation and damage to the skeleton.

Based on the analysis above, it can be concluded that the hoof cuticle can passively contribute to anti-slip and cushion of blue sheep during climbing, based on the smart combination of the micro surface morphologies, special structures of cross section and material composition. This may inspire the innovative bionic design of ground-contacting components, such as robot foot for working with irregular terrains, insoles and athletes’ shock-absorbing sneakers, etc. Also it can supply new ideas to develop biomimetic functional materials with enhanced friction, toughness and cushion properties, which can be used in packaging, structure members, functional surfaces, and so on.

## Conclusion

The study characterized the surface morphology, cross-sectional structure, material composition and mechanical properties of the stratum corneum of blue sheep’s hoof, and the contributions of these parameters to establishing contact stability and the reliability of blue sheep’s hoof were proposed. The main findings and conclusions are as follows:

• Three different types of morphologies, including stripe, straight and curved, were observed in the blue sheep’s hooves. These morphologies can improve the friction properties of the contact interface between the hoof surface and the rock or ground. Therefore, these features help enhance the anti-slip performance of the hoof as well as the contact stability during climbing.
• The different layers and material compositions in the cross section of the hoof constitute a soft-hard-soft-hard structure, which can effectively attenuate the strength of the contact force, absorb shock energy, and ensure the contact reliability and stability when the hoof collides with the ground or rock.
• Based on the above, it can be concluded that the hoof stratum corneum passively contributes to the contact stability of blue sheep during climbing based on the elegant combination of the micro surface morphologies, the unique cross-sectional structures and the material composition. These results may further our understanding of the structure and composition of natural composites and may also inspire the development of new materials or mechanical components with enhanced properties.

## Data Availability Statement

The raw data supporting the conclusions of this article will be made available by the authors, without undue reservation, to any qualified researcher.

## Author ConTributions

HK and XL were responsible for the experiments and manuscript preparation. JL, WL, and SZ participated in discussions and revisions. ZQ and LR worked as supervisors for all procedures.

## Conflict of Interest

The authors declare that the research was conducted in the absence of any commercial or financial relationships that could be construed as a potential conflict of interest.
